# Levels of Uranium in Soil and Dust and Their Associated Chemical Risks to Human Health: A Systematic Review and Meta-Analysis

**DOI:** 10.1177/11786302261430546

**Published:** 2026-08-12

**Authors:** Limbani Rodney Kalumbi, Kgomotso Lebelo, Masilu Daniel Masekameni, Wells Utembe, Derk Brouwer

**Affiliations:** 1 School of Public Health, University of the Witwatersrand, Johannesburg, South Africa; 2 School of Science and Technology, Malawi University of Business and Applied Sciences, Blantyre, Malawi; 3 Department of Development Studies, School of Social Sciences, University of South Africa, Pretoria, South Africa; 4 Toxicology Department, National Institute for Occupational Health, Johannesburg, South Africa

**Keywords:** exposure assessment, exposure routes, carcinogenic risk, non-carcinogenic risk, Joanna Briggs Institute

## Abstract

Environmental uranium contamination is a growing public health concern driven by mining, nuclear operations, phosphate fertiliser use, and improper waste disposal. These activities raise uranium levels in soil and dust, increasing human exposure and health risks. This systematic review and meta-analysis aimed to synthesise global data on uranium contamination in soil and dust, specifically evaluating carcinogenic and non-carcinogenic health risks across populations. A comprehensive literature search was conducted across Web of Science, Scopus, and PubMed databases for studies published between 2000 and 2025. Two independent reviewers screened titles, abstracts, and full texts, applying the Joanna Briggs Institute critical appraisal criteria for data extraction. Meta-analyses were performed, incorporating pooled estimates where appropriate. Narrative synthesis was applied to data unsuitable for meta-analysis. Eighteen studies from 13 countries met the inclusion criteria. The pooled mean uranium concentration was 3.49 mg/kg in soil and 5.10 mg/kg in dust. Regionally, the WHO South-East Asian Region (SEARO) reported the highest mean soil concentrations (5.26 mg/kg), while the Western Pacific Region recorded the highest dust concentrations (11.69 mg/kg). Non-carcinogenic hazard quotients ranged from 3.1 × 10^−13^ (children and adults) to 1.47 (adults), and carcinogenic risks spanned 3.54 × 10^−21^ (children) to 2.17 × 10^−9^ (adults). Uranium contamination in soil and dust varies by region, with SEARO and the Western Pacific as high-exposure areas. Risk ratios vary, influenced partly by age. Enhancing exposure assessment methods and age-specific risk evaluations is crucial for refining risk characterisation and informing public health interventions in uranium-affected areas.

## Plain Language Summary

How Uranium Content in Soil and Dust Affects Human Health: A Review of the Evidence

Aims and purpose of the research: This systematic review and meta-analysis aimed to synthesise global data on uranium contamination in soil and dust, specifically evaluating carcinogenic and non-carcinogenic health risks across populations. Background of the research: Environmental uranium contamination is a growing public health concern driven by mining, nuclear operations, phosphate fertiliser use, and improper waste disposal. These activities raise uranium levels in soil and dust, increasing human exposure and health risks. Methods and research design: To explore this issue, a systematic review and meta-analysis were conducted using studies published between 2000 and 2025. Researchers screened studies from Web of Science, Scopus, and PubMed databases, ensuring quality by applying standardised critical appraisal criteria. They pooled data where possible and used narrative synthesis for studies unsuitable for meta-analysis. The final review included 18 studies from 13 countries. Results and importance: Findings revealed a pooled mean uranium concentration of 3.49 mg/kg in soil and 5.10 mg/kg in dust, with SEARO showing the highest soil concentrations (5.26 mg/kg) and the Western Pacific recording the highest dust levels (11.69 mg/kg). Children exhibited higher health risks than adults in most studies. These results emphasise the need for improved exposure assessment methods and age-specific evaluations to refine risk characterisation and guide public health interventions in uranium-affected region.

## Introduction

Uranium is a naturally occurring radioactive element found in varying concentrations throughout the environment.^1-4^ It has 22 known isotopes, though only 3, U^234^, U^235^ and U^238^, naturally occur undisturbed in the Earth’s crust with relative abundances of 0.0055%, 0.720% and 99.2741%, respectively.^5,6^ Uranium is one of the common elements in the Earth’s crust, with an average crustal abundance of 2.7 mg/kg; most rocks typically contain 1 to 4 mg/kg^1^.^5^ It is present in all types of soil, rock, and water sources.^5,7^ Uranium in the Earth’s biosphere is derived from natural and anthropogenic sources. Rock weathering and volcanic eruptions are the primary natural sources of uranium in the environment, while human activities such as mining, ore processing, coal ash production, phosphate fertilisers, and nuclear waste also contribute.^5,8^ Nevertheless, mining has been identified as the leading human-induced source of uranium contamination in the environment, particularly in soil, dust, and water.^9-11^

Uranium deposition in soil and dust occurs through multiple pathways, including atmospheric deposition (air pathway), water runoff (water pathway), and industrial waste disposal (soil pathway). Atmospheric deposition results from the release of uranium-containing particles into the air, which then settle on soil and accumulate over time.^6^ Furthermore, water runoff transports uranium from mineral-rich rocks, mining sites, and industrial waste, depositing it in surrounding soils and contributing to localised contamination.^6,12^ Additionally, industrial emissions and waste release uranium-laden particulates that disperse and settle onto soil and dust.^11,13^ These deposition pathways contribute to the persistence and mobility of uranium in the environment,^5,14^ influencing its bioavailability and potential risks to human health from exposure.

In uranium-contaminated environments, human exposure to soil and dust occurs mainly via 3 pathways: inhalation of resuspended particulates, inadvertent ingestion, and dermal contact, particularly where the skin barrier is compromised.^5,6,9,11,15^ These routes can result in uranium accumulation in various organs, potentially leading to acute and chronic health effects, depending on the level and duration of exposure.^11,16-18^ Prolonged exposure, particularly in high-concentration areas, can increase the risk of kidney damage, lung cancer, and other long-term health issues due to uranium’s chemical toxicity and radioactivity.^11,15,16,19-21^ Moreover, the bioaccumulation of uranium in soil, dust, and other sources exacerbates human exposure, particularly among vulnerable populations such as children, pregnant women, and highly exposed communities,^22-25^ heightening the potential for adverse health outcomes over time.

Previous research on uranium levels in soil and dust has provided valuable insights into localised contamination.^10,26-28^ However, some limitations hinder a comprehensive understanding of its global distribution and associated health risks. Most studies focus primarily on regions with known uranium or industrial activities, potentially creating a geographically biased dataset.^29^ Furthermore, variations in sampling techniques, analysis methods, and reporting standards make cross-study comparisons challenging and limit the ability to assess trends over time.^30^ Additionally, there is evidence of increasing uranium production in Africa and Asia,^29,31,32^ and concerns over the suboptimal management of mine tailings from uranium mining, as well as other uranium-associated minerals, such as gold and coal.^13,33-35^ Due to these limitations, a systematic review of global data is essential to consolidate existing knowledge and identify research gaps. Furthermore, our study comprehensively synthesised global data on uranium contamination in soil and dust through a systematic review and meta-analysis, offering a robust, evidence-based understanding of the associated carcinogenic and non-carcinogenic human health risks. The study consolidated findings from diverse geographical regions and environmental contexts to provide critical insights into the extent and variability of uranium exposure. This evaluation is especially relevant for vulnerable populations residing near uranium mining, processing, or legacy contamination sites, where long-term exposure may lead to adverse outcomes such as cancer, renal dysfunction, and developmental issues. The findings serve as a valuable scientific resource for policymakers, public health officials, and environmental agencies seeking to strengthen regulatory frameworks, prioritise remediation efforts, and design health surveillance programmes to mitigate uranium-related health risks worldwide.

## Methods

This study has been reported in accordance with the Preferred Reporting Items for Systematic Reviews and Meta-Analyses (PRISMA) statement^36^ (Supplemental Table S1). The review protocol was registered with the International Prospective Register of Systematic Reviews (PROSPERO) database through a registration number (PROSPERO CRD42025645258).

### Review Question

What are the levels of uranium found in dust and soil, and how is its intake from various exposure routes related to both carcinogenic and non-carcinogenic health risks in the general population?

### Eligibility Criteria

#### Exposure

This review considered studies reporting exposure to environmental uranium, specifically in dust and soil.

#### Population

General population, excluding occupational exposure.

#### Context

This review included studies conducted in any setting involving the general population exposed to uranium, including communities and specific groups within a community and in any geographical location.

#### Outcomes

- Uranium levels (chemical concentration in soil and dust expressed in mg/kg or μg/g or μg/kg or ppm).
- The risk of non-carcinogenic health effects (expressed as a hazard quotient, HQ).
- The risk of non-carcinogenic health effects (expressed as a person’s chance of developing cancer over a lifetime, Incremental Lifetime Cancer Risk).

### Search Strategy

The search strategy focussed on peer-reviewed studies published in reputable, recognised databases. A 3-step search strategy was employed in this review. First, an initial limited search of MEDLINE (PubMed) was conducted to identify articles on the topic. In the second stage, the keywords in the titles and abstracts of relevant articles, together with index terms used to describe them, were utilised to develop a comprehensive search strategy for Web of Science, Scopus, and PubMed (Supplemental Table S2). The same search strategy, including all identified keywords and index terms, was used across all included databases. Finally, in stage 3, the reference list of all included sources of evidence was examined for additional studies.

Inclusion criteria were: (1) availability of full text; (2) evaluation of uranium concentrations in soil and dust and associated health risks; (3) published in English, either as a complete text or an abstract; (4) original observational, epidemiological, longitudinal, or cross-sectional studies, including human health risk assessments; and (5) published between 2000 and 2025. Due to the reviewer’s language limitations, only studies published or translated into English were considered for review. Review articles, letters to the editor, commentaries, replicated or unrelated studies, and literature with insufficient data were excluded.

### Study Selection

**Figure 1.**
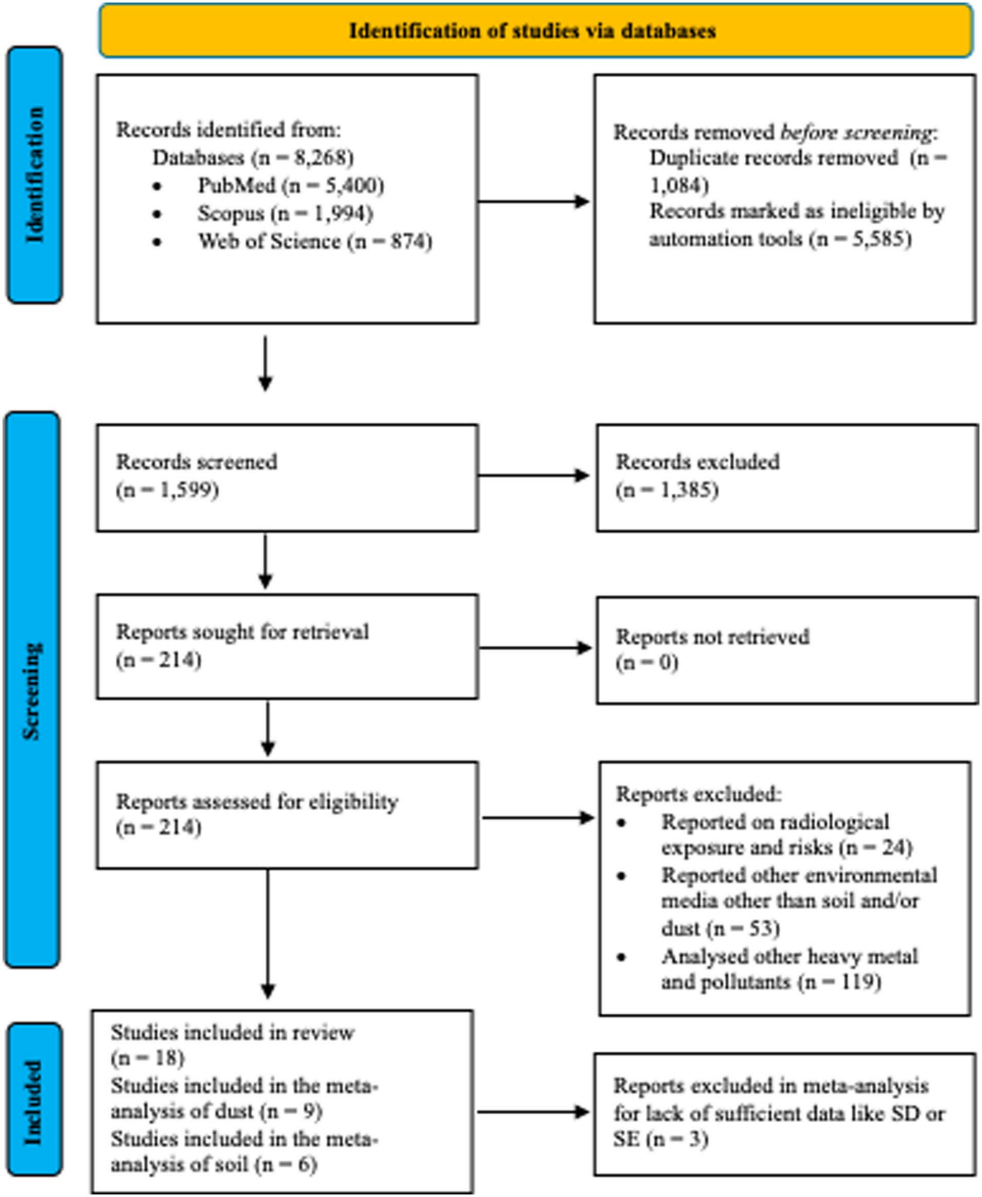
PRISMA flow diagram for identification of studies.

Following the search, all identified citations were collated and uploaded into EndNote v.21 (Clarivate Analytics, PA, USA), and duplicates were removed. Titles and abstracts were screened by 2 independent reviewers (LRK and KL) against the review’s inclusion criteria. Potentially relevant studies were retrieved in full, and their citation details were imported into the Joanna Briggs Institute (JBI) System for the Unified Management, Assessment and Review of Information (JBI SUMARI).^37^ The same reviewers independently evaluated the full text of selected citations against predefined inclusion and exclusion criteria. Eighteen studies from 13 countries worldwide met the predefined eligibility criteria and were included in the final synthesis (Figure 1). Reasons for excluding papers that did not meet the inclusion criteria were documented. Any disagreements between the reviewers at each stage of the selection process were resolved through discussion.

### Assessment of Methodological Quality

To mitigate the risk of bias, two independent reviewers (LRK and KL) critically appraised eligible studies at the study level. The studies included in this review did not directly focus on human participants but instead modelled exposure risks for both children and adults based on environmental concentrations. We were unable to identify a suitable study quality assessment tool that encompasses the range of methodologies used in the selected studies. Therefore, the quality of the studies was assessed using a modified version of the Critical Appraisal Skills Programme (CASP) checklist, which is designed for descriptive and cross-sectional studies.^38^ The CASP checklist contains questions that interrogate the validity, what the results are, and their usefulness.^39,40^ A 12-point rating system (one point for each question) was used to assess study quality (Supplementary Table S2). Scores between 10 and 12 indicated high quality, 7 and 9 were considered moderate quality, and scores of 6 and below were defined as low quality. Any disagreements were resolved through discussion among the reviewers. The critical appraisal outcomes are presented in narrative and tabular formats. Data extraction and synthesis were conducted for all included studies, regardless of their assessed methodological quality.

### Data Extraction

Data extraction was conducted after study selection and involved systematically recording predefined variables from each included study. Two independent reviewers (LRK and KL) employed standardised JBI SUMARI data extraction tools to gather data from the selected papers. The extracted data included the country, sample size, sampling area, uranium concentration, standard error or standard deviation value in dust or soil, exposure pathways, and carcinogenic and non-carcinogenic risks. The summary of extracted data is provided in Supplemental Table S4.

### Data Synthesis

Studies with sufficient data on sample sizes, means, and standard deviations or standard errors were pooled in a statistical meta-analysis using R Statistical Software (v4.5.0; R Core Team 2025). In meta-analysis using R software for single-sample studies, the mean and standard deviation, as well as the sample size, are required for continuous data.^41^ In this review, one study^42^ reported measurement error along with the mean, and Equation 1 was used to convert the measurement error to a standard deviation.^43^

(1) $$\begin{array}{l} \mathrm{Standard}\;\mathrm{Deviation}\;\left(\mathrm{SD}\right)=\mathrm{Standard}\;\mathrm{Error}\;\left(\mathrm{SE}\right)\\ \;x\;\sqrt{\mathrm{Sample}\;\mathrm{Size}\;\left(n\right)} \end{array}$$

Mean and standard deviation values reported initially in μg/g or μg/kg were converted to mg/kg using multiplication factors of 1 and 0.001, respectively. The study employed the Cochrane Q statistic to indicate the presence of heterogeneity and the *I*-squared (*I*^2^) statistic to assess the magnitude of between-study heterogeneity.^44^ Studies with *I*^2^ values greater than 50% were considered heterogeneous.^45^ The random-effects model was utilised to assess uranium concentrations in soil and dust.^45^ Publication bias was assessed using Egger’s test and graphically represented using funnel plots.^46^ Where meta-analysis was deemed inappropriate, such as the analysis of health risks, a narrative summary was prepared from the extracted data. It summarised study characteristics and data relevant to the specified review outcomes in a tabular format.

## Results

### Search Results

The initial search generated 8268 peer-reviewed articles from Scopus (1994), PubMed (5400), and Web of Science (874) published between 2000 and 2025. After collating the studies, the deduplication process identified 1084 duplicates, which were excluded. The EndNote automation system identified 5585 articles as ineligible based on their titles. Further title and abstract screening excluded 1385 articles. Subsequently, a total of 214 articles were sought for retrieval; 18 of these were included in this review, and 195 were excluded, as detailed in Figure 1.

### Study Characteristics

**Figure 2.**
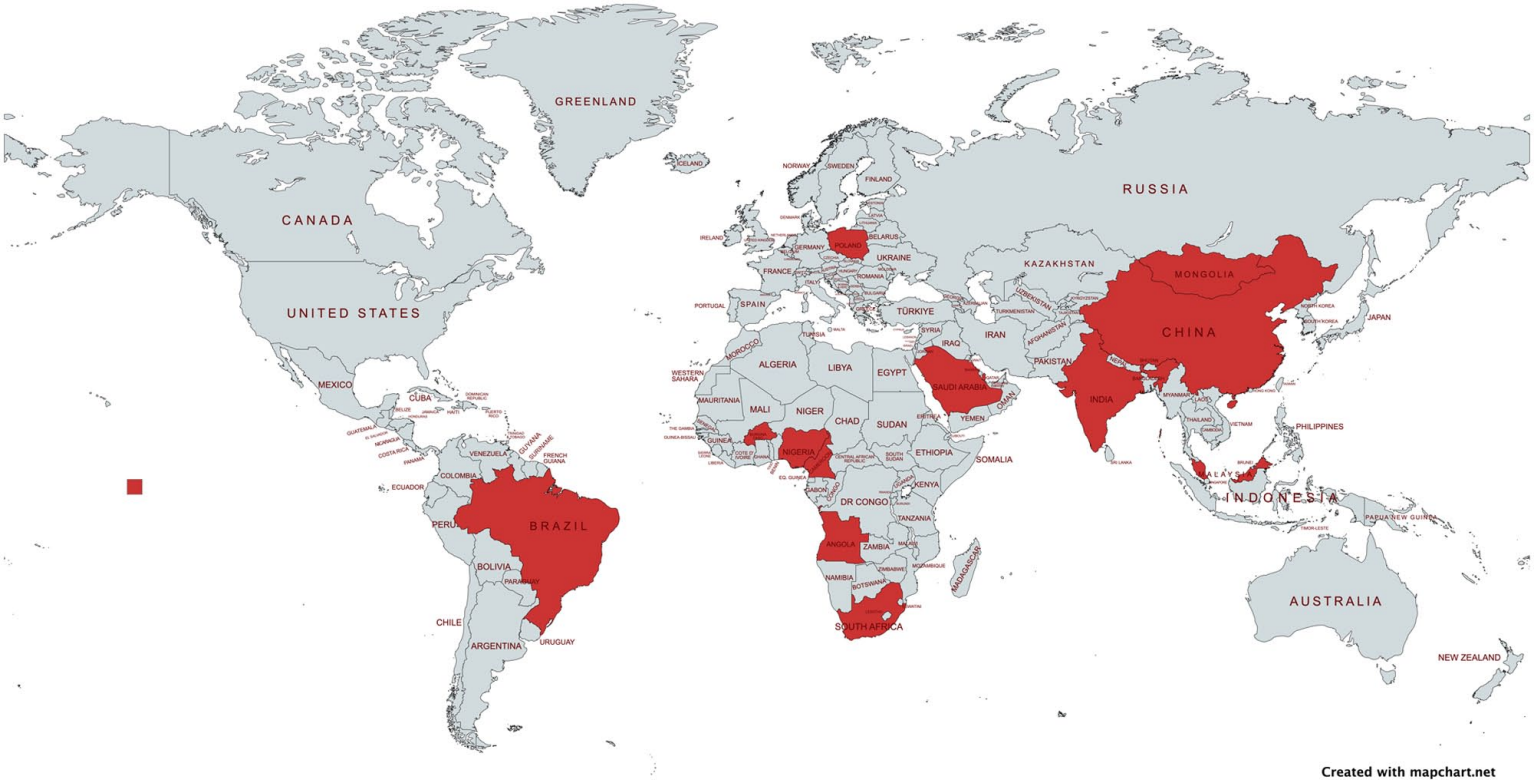
Geographical distribution of studies included.

Geographically, the studies included in this review spanned 13 countries across all the continents except North America (Figure 2). The number of countries represented in the review reflects the geographic distribution of studies that met the predefined eligibility criteria after full-text screening. When classified according to the WHO regional designation, the 18 included studies were categorised as follows: African Region (n = 6),^47-52^ Western Pacific Region (n = 4),^53-56^ Eastern Mediterranean Region (n = 4),^57-60^ South East Asian Region (SEARO, n = 2),^61,62^ Americas (n = 1),^63^ and European Region (n = 1).^42^ One study sampled both soil and dust.^55^ The number of samples analysed in the included studies varied from <30 samples (n = 7 studies), 30 to 100 samples (n = 9 studies), and >100 samples (n = 3 studies).

Active sampling methods were utilised in 14 studies (78%). The specific techniques included brush and pan (n = 8), spatula or scoop (n = 4), and shovel (n = 1). One study described the sampling approach simply as a ‘manual collector’ without further specification.^63^ Additionally, 4 studies did not report the specific materials used to collect soil or dust samples. Regarding the instrumentation used to detect uranium in the samples, nearly two-thirds (n = 11) of the studies used inductively coupled plasma mass spectrometry (ICP-MS), about one-fifth (n = 4) used energy dispersive X-ray fluorescence (ED-XRF), 2 studies used both ICP-MS and inductively coupled plasma optical emission spectroscopy (ICP-OES), and one study used field portable X-ray fluorescence (FP-XRF; Supplemental Table S3).

All the studies included in the review utilised deterministic risk assessment methods to evaluate health risks for both children and adults. Ten studies assessed potential non-carcinogenic health effects using hazard quotients (HQs). On the other hand, one study reported carcinogenic risks by quantifying the lifetime probability of developing cancer due to uranium exposure.

### Quality Assessment

Two independent reviewers assessed the quality of the studies using the modified CASP tool. Of the 18 included studies, 15 (83%) were rated high quality and 3 (17%) were rated moderate quality. Details of the CASP ratings are shown in Supplemental Table S2.

### Levels of Uranium in the Soil

**Figure 3.**
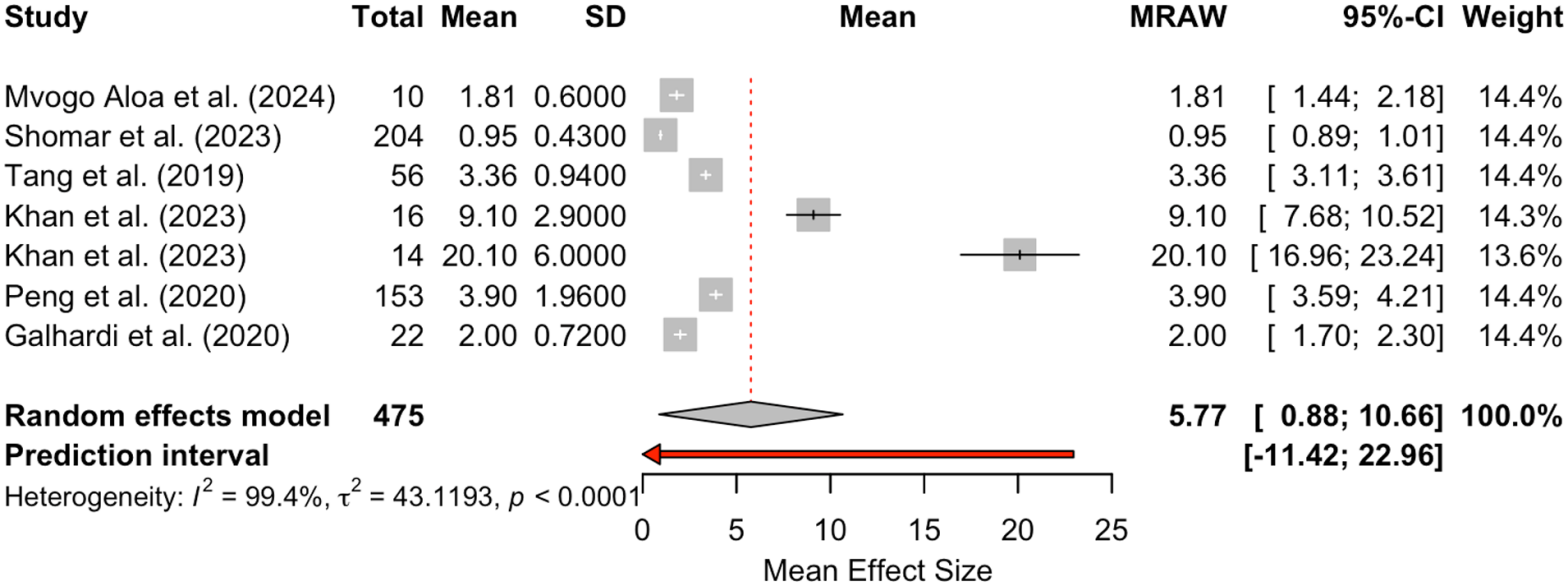
Forest plot of concentrations of uranium in soil across studies.

Figure 3 is a forest plot of uranium content from 6 studies with sufficient data to be pooled. In the figure, MRAW represents a meta-analysis of single raw (untransformed) means that utilises inverse-variance weighting to estimate a pooled mean effect across studies reporting individual means. Uranium concentrations in soils were obtained from 475 samples, ranging from 10 to 204 per study. Across the studies, uranium levels show notable variability, ranging from 0.95 mg/kg in Qatar^60^ to 20.1 mg/kg in India,^62^ with a pooled mean concentration of 5.77 mg/kg (95%CI: 0.88-10.66) under the random effects model. Notably, the high heterogeneity (*I*^2^ = 99.5%) indicates considerable dispersion in the uranium concentration of the included studies.

**Figure 4.**
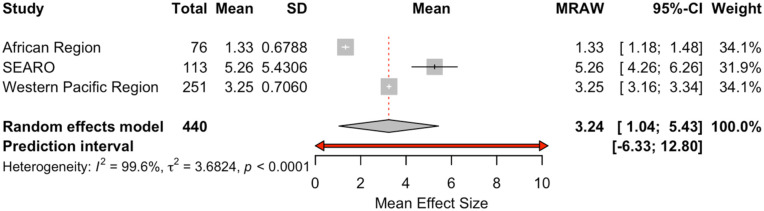
Forest plot for uranium concentrations in soil according to WHO regions.

Uranium concentrations in soil were further classified according to World Health Organisation (WHO) regions to assess geographical variability in contamination levels. Across the studies in this analysis, uranium concentrations ranged from 1.33 mg/kg in the African Region (based on 76 samples) to 5.26 mg/kg in SEARO (derived from 113 samples; Figure 4). The Western Pacific Region showed intermediate levels, contributing to a pooled mean uranium concentration of 3.24 mg/kg (95%CI: 0.88-10.66) across all 3 regions with sufficient data. Weighted mean comparisons revealed that uranium concentrations in soil samples from the African Region were 2.4 times lower than those from the Western Pacific Region (Asia) and 4.1 times lower than those from SEARO.

### Levels of Uranium in the Dust

**Figure 5.**
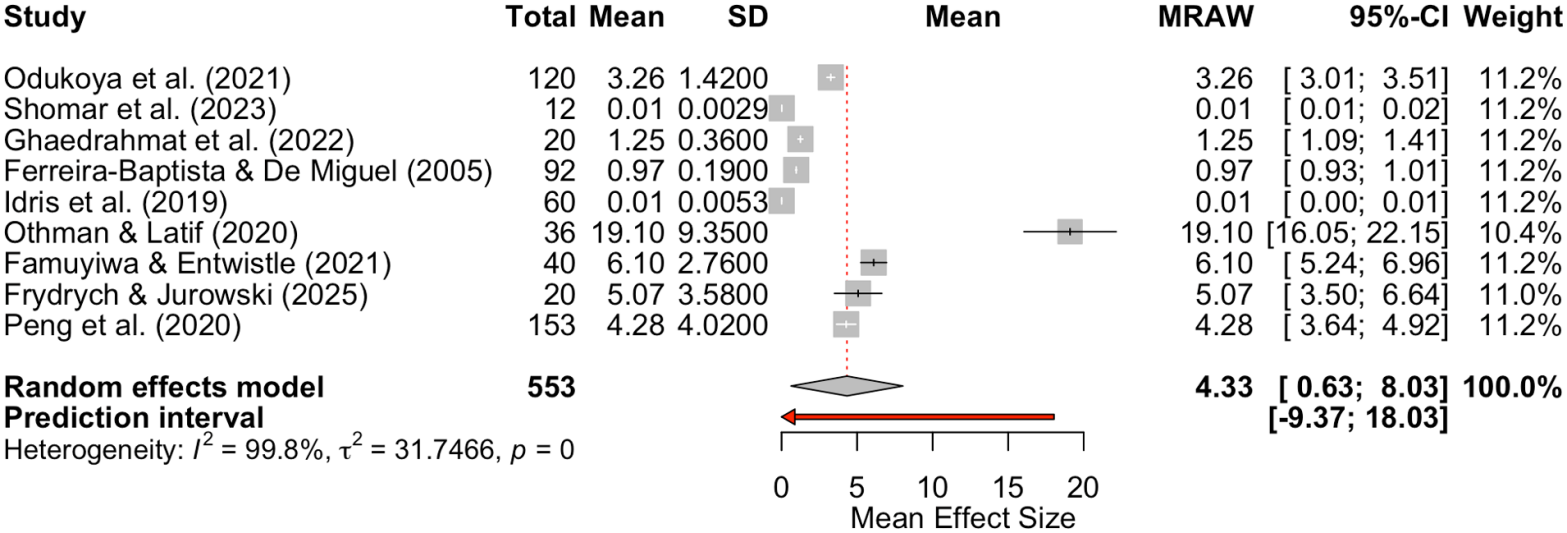
Forest plot of concentrations of uranium in dust across studies.

Nine of the 10 studies that included dust data were sufficient for the meta-analysis, allowing a pooled assessment of uranium concentrations. The number of samples analysed across these studies varied considerably, ranging from 12 to 153, yielding a cumulative total of 553 samples (Figure 5). Reported uranium concentrations showed substantial variation, ranging from 0.01in Qatar^60^ to 19.1 mg/kg in Malaysia.^54^ The pooled mean uranium concentration was estimated at 4.33 mg/kg (95%CI: 0.63-8.03) using the random-effects model. However, the statistical analysis revealed significant heterogeneity among the included studies, as indicated by an *I*^2^ value of 99.8%, suggesting considerable differences in uranium concentrations across the locations.

**Figure 6.**
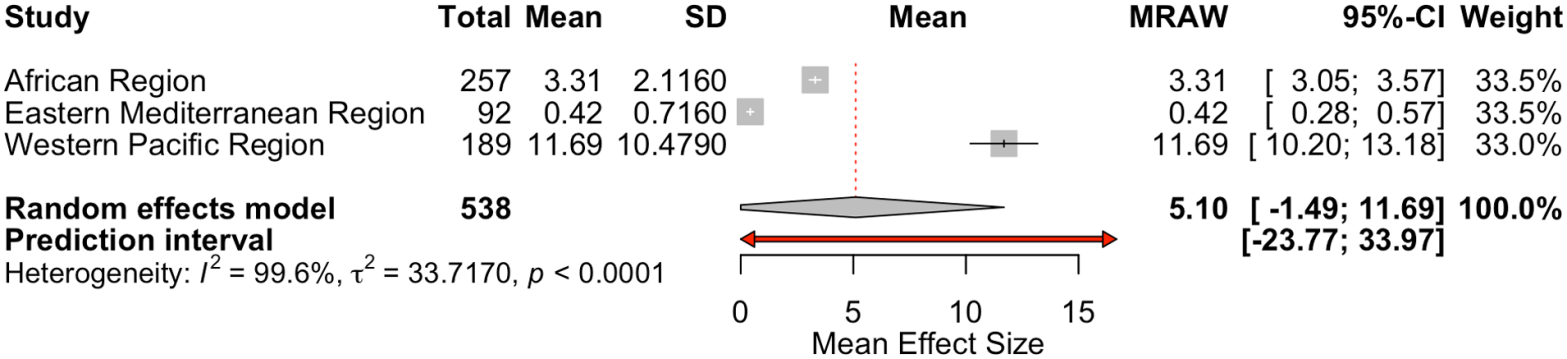
Forest plot for uranium concentrations in dust according to WHO regions.

Figure 6 is a forest plot showing uranium concentrations in dust across 3 WHO regions – the African, Eastern Mediterranean, and Western Pacific regions. Uranium levels vary substantially across these regions, with the Eastern Mediterranean Region having the lowest concentration at 0.42 mg/kg. In contrast, the Western Pacific Region had the highest concentration at 11.69 mg/kg. The random effects model estimates an overall mean uranium concentration of 5.10 mg/kg. Compared with the other 2 regions, the concentration in the Western Pacific Region was significantly higher (11.69 mg/kg, 95%CI: 10.20-13.18).

### Reported Carcinogenic and Non-carcinogenic Health Risks

Table 1 summarises reported health risks across the included studies. Ten of the 18 studies reported health risks. The findings show variations in non-carcinogenic and carcinogenic risks via ingestion, inhalation, and dermal contact pathways. Seven studies reported health risks associated with dust exposure and presented a wide range of non-carcinogenic risk estimates, particularly through inhalation and dermal exposure pathways. For instance, inhalation risks reported in Saudi Arabia (4.20 × 10^−1^) and South Africa (1.90 × 10^−1^ for children and 2.5 × 10^−1^ for adults) were higher than in other countries. For the dermal pathway, dust-related health risks were reported in South Africa (6.55 × 10^−4^ for children and 4.88 × 10^−4^ for adults) and were elevated in Iran at 1.47 in adults. By contrast, Poland^42^ reported relatively low hazard values via inhalation and dermal routes (3.10 × 10^−13^), indicating minimal risk. Ingestion risks were most pronounced in Saudi Arabia (4.20 × 10^−1^ for children and 4.5 × 10^−2^ for adults) and Poland (1.11 × 10^−2^). Three studies reported soil-related health risks. For the ingestion pathway, the risks ranged from 4.13 × 10^−3^ in adults to 3.86 × 10^−2^ in children in Cameroon. Soil-related dermal risks ranged from 1.65 × 10^−5^ among adults in Cameroon to 1.3 × 10^−4^ in Qatar. Across the studies, pathway-specific health risks indicated that ingestion was the dominant route.

**Table 1.** Non-Carcinogenic and Carcinogenic Risks Reported in the Included Studies.

Study	Country	Environmental matrix	Non-carcinogenic			Carcinogenic		
			Ingestion	Inhalation	Dermal	Ingestion	Inhalation	Dermal
Shomar & Rashkeev, 2023	Qatar	Dust	-	3.23 × 10^−7^	4.81 × 10^−8^	-	-	-
Ghaedrahmat et al., 2022	Iran	Dust	-	6.7 × 10^−1^ (Children) 3.3 × 10^−2^ (Adults)	1.47 (Adults)	-	-	-
Mvogo Aloa et al., 2024	Cameroon	Soil	3.86 × 10^−2^ (Children); 4.13 × 10^−3^ (Adults)	1.08 × 10^−6^ (Children); 6.06 × 10^−7^ (Adults)	1.08 × 10^−4^ (Children); 1.65 × 10^−5^ (Adults)	6.34 × 10^−17^ (Children); 5.44 × 10^−17^ (Adults)	3.54 × 10^−21^ (Children); 8.00 × 10^−21^	3.56 × 10^−19^ (Children); 2.17 × 10^−9^ (Adults)
Ferreira-Baptista & De Miguel, 2005	Angola	Dust	1.10 × 10^−2^	3.08 × 10^−7^	3.63 × 10^−5^	-	-	-
Idris et al., 2019	Saudi Arabia	Dust	4.20 × 10^−1^ (Children); 4.5 × 10^−2^ (Adults)	-	1.38 × 10^−4^ (Children) 2.11 × 10^−4^ (Adults)	-	-	-
Shomar et al., 2023	Qatar	Soil	6.13 × 10^−3^	-	1.3 × 10^−4^	-	-	-
Othman & Latif, 2020	Malaysia	Dust	7.1 × 10^−2^ (Children-Residential); 5.1 × 10^−4^ (Adults-Residential)	2.0 × 10^−6^ (Children-Residential); 1.1 × 10^−6^ (Adults-Residential)	2.0 × 10^−4^ (Children-Residential); 3.0 × 10^−4^ (Adults-Residential)	-	-	-
Frydrych & Jurowski, 2025	Poland	Dust	1.11 × 10^−2^	3.10 × 10^−13^	3.10 × 10^−13^	-	-	-
Mugudamani et al., 2022	South Africa	Dust	1.90 × 10^−1^ (Children); 2.5 × 10^−2^ (Adults)	-	6.55 × 10^−4^ (Children) 4.88 × 10^−4^ (Adults)	-	-	-
Galhardi et al., 2020	Brazil	Soil	0.55 × 10^−4^ (Children; Site A); 0.07 × 10^−4^ (Adults, Site A) 18 × 10^−4^ (Children; Site B), 2.3 × 10^−4^ (Adults; Site B)			-	-	-

The results show substantial variation in soil exposure risk across different age groups. Age-stratified analyses revealed that children are generally exposed to markedly higher levels. However, 2 studies in South Africa and Saudi Arabia found that adults faced greater carcinogenic risks than children. In contrast, studies conducted in Cameroon, Saudi Arabia and Brazil reported non-carcinogenic risks for children that were 9.4 times (ingestion route), 9.3 (ingestion pathway) and 7.8 times higher than those for adults, respectively. This suggests that age-related differential exposures exist. Despite the risks in select studies, values generally remained at lower magnitudes. The carcinogenic risks were reported in a study conducted in Cameroon and were found to be low.

### Publication Bias Assessment

**Figure 7.**
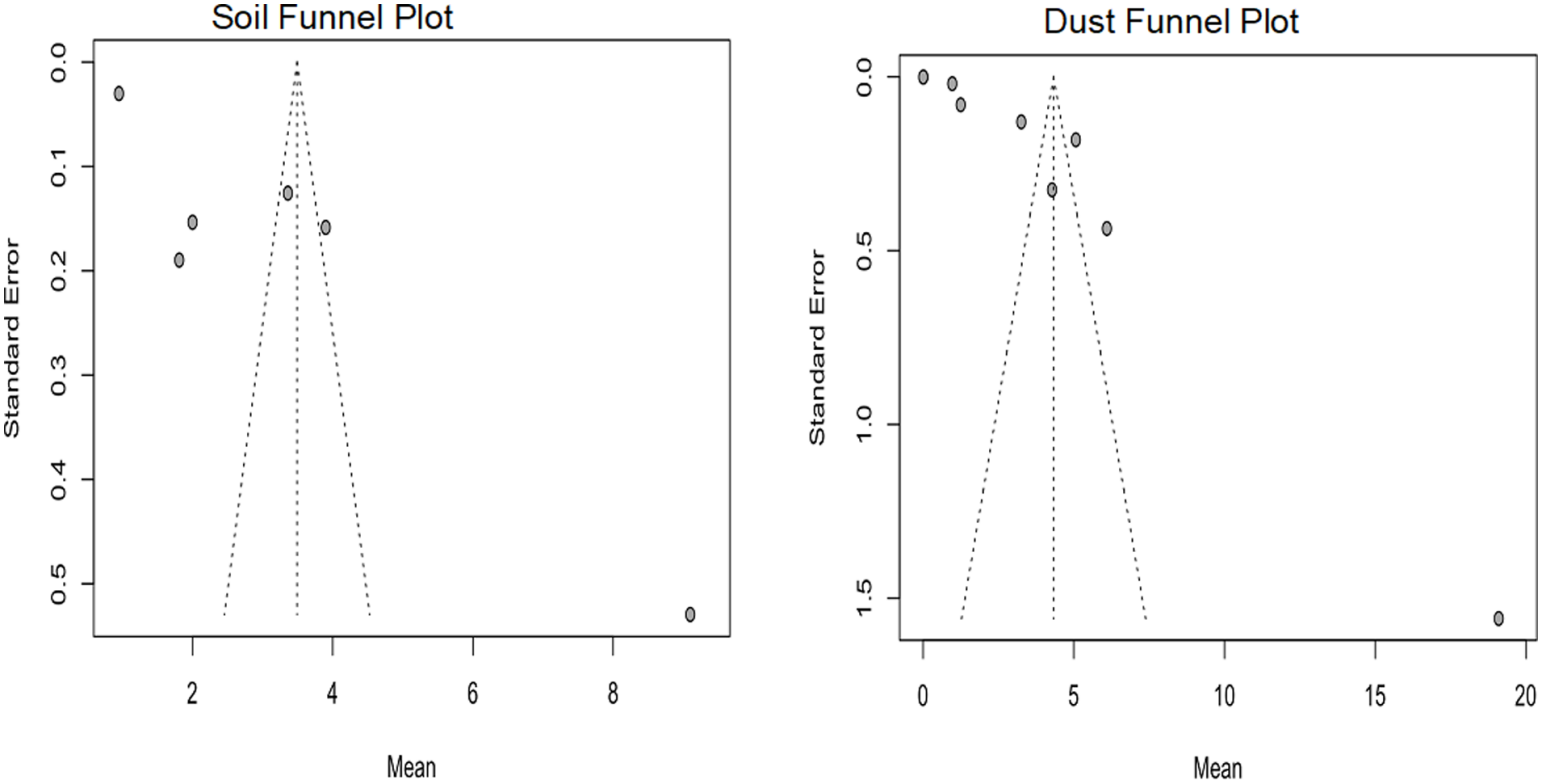
Funnel plots of uranium in soil and dust for assessing publication bias.

The study utilised funnel plots (as a visual tool) to check the efficacy of publication bias. Figure 7 presents funnel plots of mean uranium concentrations against standard errors for soil and dust studies. Because the number of included studies is small (soil: n = 6; dust: n = 9), the ability of funnel plots to detect publication bias is limited. The soil plot appears relatively symmetric, whereas the dust plot shows wider dispersion, which is more likely attributable to between-study heterogeneity in concentrations and study characteristics than to publication bias. The meta-analysis included 6 soil and 9 dust studies. Therefore, Egger’s test lacked statistical power to detect bias, as the number of studies was small, and typically, more than 10 studies are required.

## Discussion

This systematic review summarises current evidence on uranium exposure in soil and dust samples. Data reporting on uranium concentrations and their associated carcinogenic and non-carcinogenic risks varies in quantity and quality between countries, regions, and environmental matrices. Studies from only 13 countries qualified for inclusion in the review. Many countries therefore have minimal or no information on uranium concentrations in soil and dust, as well as the associated health risks. This heterogeneous distribution of currently available data indicates the need for additional research to achieve even coverage of key basic data globally.^6,13,16^ The current status quo can be attributed to the scarcity of research assessing uranium exposure as an isolated metal rather than the current situation, where it is mostly assessed along with other heavy metals. This potentially obscures uranium’s unique toxicological profile. Investigative approaches that focus solely on uranium can provide a clearer understanding of its contribution to human exposure and associated health risks. Countries which mine uranium or minerals (eg, coal, gold, silver, copper, etc.) associated with it^64^ and those with heavy industrialisation could be of particular interest in this regard.

The findings from this review indicate that tools for assessing the quality of studies to be included in systematic reviews of environmental exposures and risk assessment studies, where environmental matrices are sampled rather than human participants, are scarce. Most tools are designed for studies involving human participants, which may not be suitable for environmental studies, particularly those involving exposure assessments. This limitation is reflected in the literature, which shows a lack of such tools and indicates that most methodologies for assessing study quality have been adopted from clinical epidemiology.^65-67^ These methodologies are likely not sufficiently modified to assess and integrate evidence from environmental exposure assessments. Consequently, this compromises the quality and utility of systematic reviews in these contexts as reviewers may struggle to adequately assess study reliability, potentially introducing biases into the synthesised evidence. Addressing this gap by developing specialised appraisal tools can enhance the validity of the reviews, improve comparability across the studies, and strengthen the integration of exposure assessment evidence into the public health space.

This review has also revealed that most studies on dust exposure analysed settled dust, and none assessed airborne dust. The implications of this limitation could be significant, as airborne dust is the primary inhalation pathway. Studies have shown that inhalation of uranium particulates constitutes one of the primary routes of exposure in both occupational and environmental settings.^9,11^ The absence of airborne dust assessment limits the ability to accurately quantify exposure levels, potentially leading to underestimation of exposure levels. Future research should include airborne dust sampling to establish a more comprehensive exposure profile. Furthermore, among the studies reviewed, only one evaluated indoor dust, while the rest focussed on outdoor dust exposure. The U.S. Environmental Protection Agency has demonstrated that individuals spend a substantial portion of their time indoors,^68^ emphasising the need for future research to consider indoor contamination when conducting risk assessments.

Although the aim of the included studies was to assess the levels and health risks of multiple heavy metals, including uranium, a considerable number (8 out of 18) of the studies in the dataset reported only concentrations, thereby omitting the health risks associated with uranium. However, they did report risks for other selected metals. The rationale for reporting health risks for selected metals while omitting others was rarely articulated. Additionally, some basic parameters, particularly measures of variability (standard deviation, standard error, interquartile ranges, etc.), were missing. This omission reduces the reliability of effect size calculations, undermining the robustness of pooled estimates. The missingness in this review aligns with the literature on meta-analysis; missing data can generally be classified into 2 categories: the absence of standard deviations or sample sizes linked to the reported means and missing information on moderators.^69-71^ Studies have found missing data parameters to be a common challenge in systematic and meta-analyses in the environmental sciences.^71-73^ These findings call for greater adherence to best practices in risk assessments. Future studies should aim to provide comprehensive data on uranium exposure and its associated carcinogenic and non-carcinogenic risks. Moreover, standardised reporting of statistical parameters should be emphasised to ensure meaningful synthesis and improve the reliability of meta-analytic conclusions. At a minimum, summary statistical data should include sample size, mean plus standard deviation, and/or median plus quartiles or standard error. This will facilitate the pooling and comparing of data across different countries and regions in meta-analytic syntheses.

In this review, uranium content ranged from 0.95 to 9.10 mg/kg in soils, with a combined mean of 3.49 mg/kg. Dust samples showed a wider concentration range of 0.01 to 19.1 mg/kg, with a pooled mean of 4.33 mg/kg. These findings are notable because the pooled mean uranium concentrations in both environmental media exceed the average upper continental crustal concentration of 2.8 mg/kg. Specifically, uranium levels in soil and dust were approximately 1.2 and 1.5 times higher, respectively. These results indicate potential localised enrichment of uranium due to various activities. These findings align with previous reports documenting levels exceeding background concentrations in regions affected by mining and industrial emissions.^74,75^

Furthermore, the WHO’s regional classification of uranium concentrations highlights notable spatial variability in contamination levels, with SEARO showing higher levels than Africa and the Western Pacific. Elevated levels in the SEARO region may be attributed to a combination of natural geological factors, such as uranium-rich bedrock, and anthropogenic influences, including mining activities, industrial emissions, and the use of phosphate-based fertilisers, which are prevalent in certain parts of this region.^9,64,76^ While uranium concentrations across the regions remain within expected geological ranges, potential localised enrichment in the SEARO region may require further assessment. However, these findings should be interpreted with caution, given the limited number of studies included in the meta-analysis and the heterogeneity in study designs and measurement approaches.

Carcinogenic risk assessments, though limited in reporting across the studies, provide a picture of uranium exposure. One study in Cameroon provided carcinogenic estimates; notably, the values were low across the different pathways. On the other hand, the reported non-carcinogenic risk estimates differ across exposure pathways, with ingestion risks varying considerably among studies. For instance, the highest ingestion carcinogenic risk was observed in Saudi Arabia at 4.20 × 10^−1^, whereas it was lower in Cameroon for adults at 4.13 × 10^−3^. Likewise, inhalation risk shows heterogeneity, ranging from 6.7 × 10^−1^ in Iran to considerably lower levels in Poland at 3.10 × 10^−13^. As observed for uranium concentrations, these differences in exposure risk estimates may stem from regional disparities in soil and dust composition, driven by natural and anthropogenic activities that influence contamination levels. Dermal exposure risks further reinforce the observed differences, with the highest at 1.47 in Iran. The findings from Iran, Cameroon, Saudi Arabia, Malaysia, South Africa, and Brazil indicate differential exposure by age group, with children demonstrating higher risk than adults in most studies. These findings are consistent with research conducted in South Africa, which reported significantly higher uranium levels in children’s hair than in adults’ hair.^77^ The presence of uranium in hair indicates chronic exposure.^78,79^ Such differences emphasise the vulnerability of younger populations, suggesting the need to include age-specific risk assessments when evaluating environmental contamination in the general population.

This review considered 3 major databases (PubMed, Scopus, and Web of Science); as a result, any publications available only in other databases would have been missed. Furthermore, the search was limited to the past 2 decades (2000-2025), and we observed that little attention was given to uranium exposure and its associated human health risks prior to this period. One limitation of this study is the number of peer-reviewed articles included in the meta-analysis, which reduces statistical power and potentially increases the risk of publication bias. Publication bias cannot be excluded, as studies reporting null or low uranium concentrations may be underrepresented. Another limitation is the uneven distribution of included studies across the regions, which reduces the utility of comparisons across different countries and geographic areas. Additionally, a key limitation of this review is the heterogeneity of study methodologies and the variable completeness of data reporting across the included studies, which may affect the comparability of findings and the robustness of the synthesis. Nevertheless, this study has comprehensively synthesised dispersed evidence on uranium contamination in soil and dust, enabling more robust comparisons across regions, identifying critical data gaps, and supporting the development of context-specific exposure assessments.

## Conclusions

This systematic review was conducted to estimate uranium concentrations in soil and dust and the associated non-carcinogenic and carcinogenic risks in studies published between 2000 and 2025. It included 18 studies. Some studies had gaps in their reported data, with some parameters or outcomes missing. The average uranium content in soil and dust varies widely, with higher concentrations in some countries and regions than in the upper crust. Likewise, there are variations in non-carcinogenic and carcinogenic risks, although most are lower. However, the risks were higher in children than in adults. In the future, it is recommended that complete exposure data, including statistical parameters (sample size, mean, and standard deviation or standard error), be reported. This review recommends adopting standardised exposure assessment and reporting methods. Further, future chemical risk assessments should explicitly consider age-specific exposure scenarios to support risk characterisation and comparison across groups, such as infants, toddlers, adolescents, and adults. Finally, the review recommends expanded environmental monitoring in regions with elevated uranium concentrations to support evidence-based public health decision-making.

## Supplemental Material

sj-docx-1-ehi-10.1177_11786302261430546 – Supplemental material for Levels of Uranium in Soil and Dust and Their Associated Chemical Risks to Human Health: A Systematic Review and Meta-Analysis Supplemental material, sj-docx-1-ehi-10.1177_11786302261430546 for Levels of Uranium in Soil and Dust and Their Associated Chemical Risks to Human Health: A Systematic Review and Meta-Analysis by Limbani Rodney Kalumbi, Kgomotso Lebelo, Masilu Daniel Masekameni, Wells Utembe and Derk Brouwer in Environmental Health Insights
