# Supplementary material for: Levels of Uranium in Soil and Dust and Their Associated Chemical Risks to Human Health: A Systematic Review and Meta-Analysis
Source: Environ Health Insights. 2026 Aug 12;20:11786302261430546. doi: 10.1177/11786302261430546 (PMC13469534; doi:10.1177/11786302261430546)
Supplement: Supplementary file 1 [file sj-docx-1-ehi-10.1177_11786302261430546.docx]

## SUPPLEMENTARY INFORMATION for

## Levels of uranium in soil and dust and their associated risks to human health: a systematic review and meta-analysis

**Supplementary Table S1: PRISMA Checklist**

| **Section and Topic** | **Item #** | **Checklist item** | **Location where item is reported** |
| --- | --- | --- | --- |
| **TITLE** | | |  |
| Title | 1 | Levels of uranium in soil and dust and their associated risks to human health: a systematic review and meta-analysis | Title |
| **ABSTRACT** | | |  |
| Abstract | 2 | Provide a structured summary including, as applicable: background; objectives; data sources; study eligibility criteria, participants, and interventions; study appraisal and synthesis methods; results; limitations; conclusions and implications of key findings; systematic review registration number. | Pg 1 |
| **INTRODUCTION** | | |  |
| Rationale | 3 | Describe the rationale for the review in the context of existing knowledge. | Pg 2-4 |
| Objectives | 4 | Provide an explicit statement of the objective(s) or question(s) the review addresses. | Pg 4 |
| **METHODS** | | |  |
| Eligibility criteria | 5 | Specify the inclusion and exclusion criteria for the review and how studies were grouped for the syntheses. | Pg 5 |
| Information sources | 6 | Specify all databases, registers, websites, organisations, reference lists and other sources searched or consulted to identify studies. Specify the date when each source was last searched or consulted. | Pg 5 |
| Search strategy | 7 | Present the full search strategies for all databases, registers and websites, including any filters and limits used. | Supplementary Table X |
| Selection process | 8 | Specify the methods used to decide whether a study met the inclusion criteria of the review, including how many reviewers screened each record and each report retrieved, whether they worked independently, and if applicable, details of automation tools used in the process. | Pg 6 |
| Data collection process | 9 | Specify the methods used to collect data from reports, including how many reviewers collected data from each report, whether they worked independently, any processes for obtaining or confirming data from study investigators, and if applicable, details of automation tools used in the process. | Pg 6 |
| Data items | 10a | List and define all outcomes for which data were sought. Specify whether all results that were compatible with each outcome domain in each study were sought (e.g. for all measures, time points, analyses), and if not, the methods used to decide which results to collect. | Pg 5 |
|  | 10b | List and define all other variables for which data were sought (e.g. participant and intervention characteristics, funding sources). Describe any assumptions made about any missing or unclear information. | Pg 5 |
| Study risk of bias assessment | 11 | Specify the methods used to assess risk of bias in the included studies, including details of the tool(s) used, how many reviewers assessed each study and whether they worked independently, and if applicable, details of automation tools used in the process. | Pg 7 |
| Effect measures | 12 | Specify for each outcome the effect measure(s) (e.g. risk ratio, mean difference) used in the synthesis or presentation of results. | Not applicable |
| Synthesis methods | 13a | Describe the processes used to decide which studies were eligible for each synthesis (e.g. tabulating the study intervention characteristics and comparing against the planned groups for each synthesis (item #5)). | Pg 8 |
|  | 13c | Describe any methods used to tabulate or visually display results of individual studies and syntheses. |  |
|  | 13d | Describe any methods used to synthesize results and provide a rationale for the choice(s). If meta-analysis was performed, describe the model(s), method(s) to identify the presence and extent of statistical heterogeneity, and software package(s) used. |  |
| Reporting bias assessment | 14 | Describe any methods used to assess risk of bias due to missing results in a synthesis (arising from reporting biases). | Pg 7 |
| **RESULTS** | | |  |
| Study selection | 16a | Describe the results of the search and selection process, from the number of records identified in the search to the number of studies included in the review, ideally using a flow diagram. | Pg 8 |
|  | 16b | Cite studies that might appear to meet the inclusion criteria, but which were excluded, and explain why they were excluded. | Pg 9 |
| Study characteristics | 17 | Cite each included study and present its characteristics. | Pg 10 |
| Risk of bias in studies | 18 | Present assessments of risk of bias for each included study. | Pg 11 |
| Results of individual studies | 19 | For all outcomes, present, for each study: (a) summary statistics for each group (where appropriate) and (b) an effect estimate and its precision (e.g. confidence/credible interval), ideally using structured tables or plots. | Supplementary Table S1 |
| Results of syntheses | 20a | For each synthesis, briefly summarise the characteristics and risk of bias among contributing studies. | Pg 11-16 |
|  | 20b | Present results of all statistical syntheses conducted. If meta-analysis was done, present for each the summary estimate and its precision (e.g. confidence/credible interval) and measures of statistical heterogeneity. If comparing groups, describe the direction of the effect. |  |
| Reporting biases | 21 | Present assessments of risk of bias due to missing results (arising from reporting biases) for each synthesis assessed. | Pg 17 |
| **DISCUSSION** | | |  |
| Discussion | 23a | Provide a general interpretation of the results in the context of other evidence. | Pg 17-22 |
|  | 23b | Discuss any limitations of the evidence included in the review. |  |
|  | 23c | Discuss any limitations of the review processes used. |  |
|  | 23d | Discuss implications of the results for practice, policy, and future research. |  |
| **OTHER INFORMATION** | | |  |
| Registration and protocol | 24a | Provide registration information for the review, including register name and registration number, or state that the review was not registered. | Pg 4 |
|  | 24b | Indicate where the review protocol can be accessed, or state that a protocol was not prepared. | Registered in PROSPERO. (PROSPERO CRD42025645258) |
|  | 24c | Describe and explain any amendments to information provided at registration or in the protocol. | None |
| Support | 25 | Describe sources of financial or non-financial support for the review, and the role of the funders or sponsors in the review. | Pg 23 |
| Competing interests | 26 | Declare any competing interests of review authors. | Pg 23 |
| Availability of data, code and other materials | 27 | Report which of the following are publicly available and where they can be found: template data collection forms; data extracted from included studies; data used for all analyses; analytic code; any other materials used in the review. | Pg 23 |

**Supplementary Table S2: Search strategies for PubMed, Web of Science, and Scopus**

| **Database** | **PubMed (Medline)** |  |
| --- | --- | --- |
| **Date** | **28/03/2025** |  |
| **Strategy** | **#1 AND #2 AND #3 AND #4** |  |
| #1 | ("uranium"[All Fields] OR "uranium 238"[All Fields] OR "uranium 235"[All Fields] OR "uranium 234"[All Fields] OR "U"[All Fields] OR "U-234"[All Fields] OR "U-235"[All Fields] OR "U-238"[All Fields] OR "depleted uranium"[All Fields] OR "natural uranium"[All Fields] OR "uranium ore"[All Fields]) |  |
|  |  |  |
|  |  |  |
|  |  |  |
| #2 | ("dust"[All Fields] OR "particulate matter"[All Fields] OR "settled dust"[All Fields] OR "airborne dust"[All Fields] OR "PM"[All Fields] OR "PM2.5"[All Fields] OR "PM10"[All Fields] OR "aerosols"[All Fields] OR "fugitive dust"[All Fields] OR "indoor dust"[All Fields] OR "outdoor dust"[All Fields] OR "soil"[All Fields] OR "soil media"[All Fields] OR "soil matrix"[All Fields]) |  |
|  |  |  |
|  |  |  |
|  |  |  |
|  |  |  |
| #3 | ("concentration"[All Fields] OR "level"[All Fields] OR "amount"[All Fields] OR "content"[All Fields] OR "magnitude"[All Fields] OR "dose"[All Fields] OR "ppm"[All Fields] OR "mg/kg"[All Fields] OR "μg/kg"[All Fields]) |  |
|  |  |  |
|  |  |  |
| #4 | ("human health"[All Fields] OR "risk assessment"[All Fields] OR "human health risk assessment"[All Fields] OR "HHRA"[All Fields] OR "carcinogenic risk"[All Fields] OR "non-carcinogenic risk"[All Fields] OR "carcinogenicity"[All Fields] OR "carcinogenic risk"[All Fields] OR "risk characterization"[All Fields] OR "hazard identification"[All Fields] OR "dose-response assessment"[All Fields] OR "health hazard assessment"[All Fields] OR "toxicological risk assessment"[All Fields])) AND ((fha[Filter]) AND (2000/1/1:2025/1/1[pdat])) |  |
|  |  |  |
|  |  |  |
|  |  |  |
|  |  |  |
|  |  |  |
|  |  |  |
| **Database** | **Web of Science** |  |
| **Date** | **28/03/2025** |  |
| **Strategy** | **#1 AND #2 AND #3 AND #4** |  |
| #1 | ("uranium" OR "uranium 238" OR "uranium 235" OR "uranium 234" OR "U" OR "U-234" OR "U-235" OR "U-238" OR "depleted uranium" OR "natural uranium" OR "uranium ore") |  |
|  |  |  |
|  |  |  |
|  |  |  |
| #2 | ("dust" OR "particulate matter" OR "settled dust" OR "airborne dust" OR "PM" OR "PM2.5" OR "PM10" OR "aerosols" OR "fugitive dust" OR "indoor dust" OR "outdoor dust" OR "soil" OR "soil media" OR "soil matrix") |  |
|  |  |  |
|  |  |  |
|  |  |  |
| #3 | ("concentration" OR "level" OR "amount" OR "content" OR "magnitude" OR "dose" OR "ppm" OR "mg/kg" OR "μg/kg") |  |
|  |  |  |
|  |  |  |
| #4 | ("human health" OR "risk assessment" OR "human health risk assessment" OR "HHRA" OR "carcinogenic risk" OR "non-carcinogenic risk" OR "carcinogenicity" OR "carcinogenic risk" OR "risk characterization" OR "hazard identification" OR "dose-response assessment" OR "health hazard assessment" OR "toxicological risk assessment") |  |
|  |  |  |
|  |  |  |
|  |  |  |
|  |  |  |
|  |  |  |
| **Database** | **Scopus** |  |
| **Date** | **28/03/2025** |  |
| **Strategy** | **#1 AND #2 AND #3 AND #4** |  |
| #1 | ( "uranium" OR "uranium 238" OR "uranium 235" OR "uranium 234" OR "U" OR "U-234" OR "U-235" OR "U-238" OR "depleted uranium" OR "natural uranium" OR "uranium ore" ) |  |
|  |  |  |
|  |  |  |
|  |  |  |
| #2 | ( "dust" OR "particulate matter" OR "settled dust" OR "airborne dust" OR "PM" OR "PM2.5" OR "PM10" OR "aerosols" OR "fugitive dust" OR "indoor dust" OR "outdoor dust" OR "soil" OR "soil media" OR "soil matrix" ) |  |
|  |  |  |
|  |  |  |
|  |  |  |
| #3 | ( "concentration" OR "level" OR "amount" OR "content" OR "magnitude" OR "dose" OR "ppm" OR "mg/kg" OR "μg/kg" ) |  |
|  |  |  |
|  |  |  |
| #4 | ( "human health" OR "risk assessment" OR "human health risk assessment" OR "HHRA" OR "carcinogenic risk" OR "non-carcinogenic risk" OR "carcinogenicity" OR "carcinogenic risk" OR "risk characterization" OR "hazard identification" OR "dose-response assessment" OR "health hazard assessment" OR "toxicological risk assessment" ) ) AND PUBYEAR > 1999 AND PUBYEAR < 2026 |  |
|  |  |  |
|  |  |  |
|  |  |  |

Supplementary Table S3: Methodological Quality of the Included Studies

| **#** | **Study** | **Did the study address a clearly focused question?** | **Was the study methodologically sound?** | **Were the environmental samples clearly defined?** | **Are the sample collection and storage procedures clearly described?** | **Are the sampling sites and samples adequate?** | **Were the exposure measurements reliable?** | **Does the study acknowledge limitations and biases?** | **Did the study obtain ethical/regulatory clearance or inclusion of ethics statement?** | **Were the statistical analyses appropriate?** | **Were the results clearly presented?** | **Are the findings applicable to a broader population?** | **Were the conclusions supported by the results?** | **Score (Out of 12)** |
| --- | --- | --- | --- | --- | --- | --- | --- | --- | --- | --- | --- | --- | --- | --- |
| 1 | Battsengel et al., 2020. | Yes | Yes | Yes | Yes | Yes | Yes | No | No | Yes | Yes | Yes | Yes | 10 |
| 2 | Galhardi et al., 2020. | Yes | Yes | Yes | Yes | Yes | Yes | No | Can't Tell | Yes | Yes | Yes | Yes | 10 |
| 3 | Mugudamani et.al., 2022. | Yes | Yes | Yes | Yes | Can't Tell | Yes | No | No | Yes | Yes | Yes | Yes | 9 |
| 4 | Peng et al., 2020. | Yes | Yes | Yes | Yes | Yes | Yes | Yes | No | Yes | Yes | Yes | Yes | 11 |
| 5 | Frydrych & Jurowski, 2025. | Yes | Yes | Yes | Yes | Yes | Yes | Yes | Yes | Yes | Yes | Yes | Yes | 12 |
| 6 | Famuyiwa & Entwistle, 2021. | Yes | Yes | Yes | Yes | Yes | Yes | No | No | Yes | Yes | Yes | Yes | 10 |
| 7 | Othman & Latif, 2020. | Yes | Yes | Yes | Yes | Yes | Yes | No | No | Yes | Yes | Yes | Yes | 10 |
| 8 | Khan et al., 2023. | Yes | Yes | Yes | Yes | Yes | Yes | No | Yes | Yes | Yes | Yes | Yes | 11 |
| 9 | Tang et al., 2019. | Yes | Yes | Yes | Can't Tell | Yes | Yes | No | No | Yes | Yes | Yes | Yes | 9 |
| 10 | Shomar et. al. 2023. | Yes | Yes | Yes | Can't Tell | Yes | Yes | No | Yes | Yes | Yes | Yes | Yes | 10 |
| 11 | Idris et al., 2019. | Yes | Yes | Yes | Yes | Yes | Yes | No | No | Yes | Yes | Yes | Yes | 10 |
| 12 | Ferreira-Baptista &, De Miguel, 2005. | Yes | Yes | Yes | Yes | Yes | Yes | Yes | No | Yes | Yes | Yes | Yes | 11 |
| 13 | Mvogo Aloa et al., 2024. | Yes | Yes | Yes | Yes | Can't Tell | Yes | No | Yes | Yes | Yes | Yes | Yes | 10 |
| 14 | Ghaedrahmat et al., 2022. | Yes | Yes | Yes | Yes | Can't Tell | Yes | No | Yes | Yes | Yes | Yes | Yes | 10 |
| 15 | Coulibaly & Sako, 2025. | Yes | Yes | Yes | Can't Tell | Yes | Yes | No | No | Yes | Yes | Yes | Yes | 9 |
| 16 | Gopal et al., 2024. | Yes | Yes | Yes | Yes | Yes | Yes | No | No | Yes | Yes | Yes | Yes | 10 |
| 17 | Odukoya et al., 2021. | Yes | Yes | Yes | Yes | Yes | Yes | No | No | Yes | Yes | Yes | Yes | 10 |
| 18 | Shomar & Rashkeev, 2023. | Yes | Yes | Yes | Yes | Can't Tell | Yes | No | Yes | Yes | Yes | Yes | Yes | 10 |

Supplementary Table S4: Characteristics of Included Studies

| **Number** | **Study** | **Country** | **Year for data collection** | **Environmental Matrix** | **Type of soil or dust** | **Number of samples and measurement methods** | **Description of main results** |
| --- | --- | --- | --- | --- | --- | --- | --- |
| 1 | Battsengel E, Murayama T, Fukushi K, Nishikizawa S, Chonokhuu S, Ochir A, et al. 2020. | Mongolia | Not specified | Soil | Top soil | A total of 42 soil samples were collected were collected using a plastic spatula. Elemental analysis of 26 heavy metals, including uranium, was done by ICP-OES and ICP-MS methods. | The mean content of uranium in the samples was 2.5 mg/kg. No SD or SE. Health risks were reported in 8 out of 26 metals. For uranium, it was not reported. |
| 2 | Galhardi JA, de Mello JWV, Wilkinson KJ. 2020. | Brazil | Not specified | Soil | Farming soil | Two kg of soil samples (n=22) were collected at a depth of 20 cm using a manual collector and then stored in plastic containers. Elemental concentration was determined by ICP-MS. | The mean uranium content in the soil samples was 2.0 ± 0.7 mg/kg. The non-carcinogenic risks in children and adults were 0.55x10-4 and 0.07x10-4, respectively, at one site (Figueira) and 18x10-4 and 2.3x10-4 at another (Pocos de Caldas). Note that the study reported daily intake separately, but reported combined hazard ratio. |
| 3 | Mugudamani I, Oke SA, Gumede TP. 2022. | South Africa | 2021 | Dust | Road dust | Five (5) dust samples were collected by sweeping with a brush and dustpan. The concentration of major and trace elements, including uranium, in dust, was determined using the wavelength-dispersive XRF method. | The average mean concentration of uranium was 2.9 mg/kg ± 0.0. The HQ for the ingestion pathway was 1.90x10-1 for children and 2.5x10-2 for adults, and for the dermal pathway was 6.55x10-4 for children and 4.88x10-4 for adults. |
| 4 | Peng L, Li X, Sun X, Yang T, Zhang Y, Gao Y, et al. 2020. | China | 2014 | Dust and Soil | Street dust and urban soil | A total of 458 samples, including 153 urban soil, 153 street dust, and 152 road plant samples, were collected. Metal content in soil and dust samples were determined by X-Ray Fluorescence spectrometer. | The uranium content in the soil and dust were 3.90 ± 1.96 mg/kg and 4.28 ± 4.02 mg/kg. Carcinogenic and non-carcinogenic risks for uranium were not reported, though health risks were reported in 10 out of 17. |
| 5 | Frydrych A, Jurowski K. 2025. | Poland | Not specified | Dust | Road dust | Samples of road dust (RD) from petrol stations (n = 20).Clear zip-lock bags were held close to the ground to collect the samples, and a sufficient amount of RD was collected using a hand brush. The determination of the concentration of elements was done by applying the FP-XRF spectrometer. | The mean uranium concentration in the dust was 5.07± 0.81 µg/g. The HQs for the ingestion, inhalation, and dermal pathways were 1.11x10−2, 3.10x10−13, and 3.10x10−13, respectively. |
| 6 | Famuyiwa AO, Entwistle JA. 2021. | Nigeria | 2019 | Dust | Settled indoor dust | Classroom dust samples were collected from 40 locations across the twenty local government areas. Classroom dust was collected using a combination of short sweeping brushes and/or long-handled brooms to sweep together dust from across the sampled room. Elemental concentration analysis was done by ICP-MS. | The mean uranium concentration in the sampled dust was 6.1 ± 2.76 mg/kg. The cancer and non-cancer risks were reported in other elements. |
| 7 | Othman M, Latif MT. 2020. | Malaysia | 2018 | Dust | Road dust | A total of 36 road dust samples from five different urban areas - commercial, high traffic, park, rail station, and residential areas - were collected using plastic dustpans and brushes. Elemental concentration analysis was done by ICP-MS. | The overall uranium average in the soil samples was 19.1 ± 9.35 μg/g. The residential non-cancer risks for the inhalation, dermal and ingestion routes were 2.0x10-6, 2.0x10-4 and 7.1x10-2, respectively, in children, whereas in adults, they were 1.1x10-6, 3.0x10-4, and 5.1x10-4, respectively. |
| 8 | Khan I, Choudhary BC, Izhar S, Kumar D, Satyanarayanan M, Rajput VD, et al. 2023. | India | 2021 | Soil | Farming soil and wetland soil | Thirty surface soil samples (n=16, wetland and n=14 farmland) were collected using using a trowel and spatula. Elemental concentrations were measured using the high-resolution ICP-MS. | The mean uranium concentration in the sampled soils was reported to be 9.1 ± 2.1 mg/kg for wetland soil and 20.1 ± 6.0 mg/kg for farming/agricultural soil. The health risks were reported in other elements, bar uranium. |
| 9 | Tang ZH, Ouyang TP, Li MK, Huang NS, Kuang YQ, Hu Q, et al. 2019. | China | Not specified | Soil | Top (surface) soil | Soil samples were collected from 56 sampling sites, mainly distributed near the residential areas. Concentrations of trace elements, including uranium, were determined using ICP-MS. | The elemental uranium concentration in the soil samples was 3.36 ± 0.94 mg/kg. Health risks were reported in five of the 21 elements, uranium was among those excluded. |
| 10 | Shomar B, Sankaran R, Solano JR. 2023. | Qatar | 2019-2020 | Soil | Top soil | Ten subsamples were collected from open, grass, vegetable, and ploughed soils and mixed to form composite samples (n=204). The samples were analysed by inductively coupled plasma-optical emission spectrometry (ICP/OES) and ICP-MS. | The average concentration in the composite soil samples was 0.95 ± 0.43mg/kg. The non-carcinogenic risks for the ingestion and dermal routes were 6.13x10-3 and 1.3x10-4, respectively. |
| 11 | Idris AM, Said TO, Brima EI, Sahlabji T, Alghamdi MM, El-Zahliar AA, et al. 2019. | Saudi Arabia | Not specified | Dust | Street dust | Sixty soil samples were collected from 20 sites using a clean polyethene shovel and brush. Elemental concentration analysis was done by ICP-MS. | The mean concentration of uranium in the dust samples 5.75 ± 5.34 μg/kg. The non-cancer risks in the ingestion and pathway were 4.20x10-1 in children and 4.5x10-2 in adults, and in the dermal route 1.38x10-4 in children and 2.11x10-4 in adults. |
| 12 | Ferreira-Baptista L, De Miguel E. 2005. | Angola | 2002 | Dust | Street dust | A total of 92 samples of street dust were collected using a plastic dustpan and brush and analysed by ICP-MS for 35 elements after an aqua-regia digestion. | The mean concentration of uranium in the samples was 0.97 ± 0.19 μg g−1. The non-carcinogenic risks for the ingestion, inhalation, and dermal routes were 1.10x-2, 3.08x10-7, and 3.63x10-5, respectively. |
| 13 | Mvogo Aloa B, Beyala Ateba JF, Gondji DS, Sabouang JF, Guembou Shouop CJ, Ema'a Ema'a JM, et al. 2024. | Cameroon | Not specified | Soil | Farming soil | Ten (10) soil samples were collected from farmer’s fields. The elemental composition analysis of soil samples was carried out using the EDXRF spectrometer. | The mean concentration of uranium in soil samples was 1.81±0.6 mg/kg. The non-carcinogenic risks in the ingestion, dermal, and inhalation pathways were 3.86x10-2, 1.08x10-4, and 1.08x1-8, respectively, in children. The non-cancer risks via the ingestion, dermal, and inhalation pathways were 4.13x10-3, 1.65x10-5, and 6.06x10-7, respectively, in adults. The cancer risks in children through the same pathways were 6.34x10-17, 3.56x10-19, and 3.54x10-21. Likewise, in adults, the carcinogenic risks in the same pathways were 5.44x10-17, 2.17x10-9, and 8.00x10-21. |
| 14 | Ghaedrahmat Z, Almasi H, Akhbarizadeh R, Ahmadi M. 2022. | Iran | 2019 | Dust | Road dust | A total of 20 samples were collected from main roads with different land uses, including residential, industrial, and commercial areas, using a plastic sander and plastic brush. Concentrations of metals were analysed using ICP/MSS. | The mean concentration of uranium in the samples was 1.25 ± 0.36 mg/kg. The HQ for the ingestion pathway was 6.7x10-1 in children; the HQs for the inhalation pathway were 6.7x10-1 in children and 3.3x10-2 in adults, and for the dermal pathaway it was 1.47, in adults. |
| 15 | Coulibaly K, Sako A. 2025. | Burkina Faso | Not specified | Soil | Top soil | A total of 66 topsoil samples (0–30 cm) were randomly collected using a clean stainless-steel scoop. Concentrations of 37 elements were determined using ICP-MS. | The mean uranium concentration in the soil samples was 0.85 mg/kg. No SD or SE provided. Health risks were reported for 8 of the 37 elements analysed, but uranium was among the elements that were not considered. |
| 16 | Gopal V, Krishnamurthy RR, Indhumathi A, Sharon BTX, Priya TSD, Rathinavel K, et al. 2024. | India | 2019 | Soil | Road dust | Topsoil samples (n=83) were collected using a high-density plastic spatula. The elemental concentration analysis used the Spectro XEPOS model and energy-dispersive X-ray (ED-XRF). | The mean concentration of uranium was 1.42 ± 0.9 ppm. The carcinogenic and non-carcinogenic risks were reported for eight out of eleven elements analysed; uranium health risks were among those not reported. |
| 17 | Odukoya AM, Akinwunmi SM, Watts MJ. 2021. | Nigeria | 2020 | Dust | Road dust | Urban road dust samples (n=120) were collected using a polyethene brush and plastic packer. Road dust digests were analysed by ICP-MS to determine the concentration of elements. | The mean uranium concentration in the road dust samples was 3.26 ± 1.42mg/kg. The health risks for eight of 16 were reported; uranium was among those elements whose risks were not reported. |
| 18 | Shomar B, Rashkeev SN. 2023. | Qatar | 2019-2020 | Dust | Settled dust | Dust samples (n=12) were manually collected using a soft plastic brush. Concentrations of total elements (metals and metalloids) were determined via ICP-MS. | The mean concentrations were measured over a year and reported monthly. The mean concentration for uranium was 15.3 ± 2.9 μg/kg (considered the month of July since it was the highest and mid-year). The HQ for the inhalation was 3.23x10-7, and for dermal, it was 4.81x10-8. |
